# Dr. Gregory on Small-pox

**Published:** 1844-02

**Authors:** 


*Dr. Gregory on Small-Pox.*—[The following remarks are taken from a notice of Dr. Gregory's Lecture on the Eruptive Fevers, in the London Medical Gazette, Oct. 27, 1843. Dr. G. has seen more of small-pox than any other practitioner of the present day, and consequently his opinions are entitled to great weight.—*Med. Ex.*]

The miasm, as it were, poisons the blood, altering its properties and power of coagulating in a remarkable manner. Among other instances of this the author mentions that he was last year called into consultation with Dr. L. Stewart, regarding a lady in small-pox, whose whole body was of the color of indigo, so that he took her at first for an African. She conversed with our author quite tranqilly but a few hours before her death, "proving that the nervous system is not necessarily, nor is it even usually, implicated in the petechial form of small-pox."

In the cases of adult females, Dr. Gregory states, that when thus attacked menorrhagia is almost always present, and if they be pregnant, they almost invariably miscarry, the fœtus dying in utero. This is what used to be called black small-pox by the older writers.

Ophthalmia is a very common accompaniment of small-pox; but it is a mistake to suppose (as is often done) that this arises from the formation of pustules on the cornea and conjunctiva. There is no specific inflammation of the eye in small pox, but the ophthalmia, so destructive of vision, is a sequela of the disease. It frequently sets in about the tenth day, and runs its course so rapidly that the eye is destroyed within forty-eight hours.

The chest also is frequently affected; but the abdomen is peculiarly exempt from inflammation in small-pox; thus we are told the idea of anything like pustules being found on the mucous membrane of the stomach or bowels is erroneous, the appearances in reality being "in all respects" the same as are met with in typhus fever.

"The whole human race, with very few exceptions, is susceptible of this disease, and the inhabitants of different regions seem to be in this respect on an equal footing. To this general rule there are several exceptions, and persons have been known to go through life without ever taking the disease, though frequently exposed to it. Again, some persons appear unsusceptible of the disease at one time, and yet take it at another. Dr. Gregory gives an example of this: A lady residing at Salisbury was inoculated for small-pox, in 1804, at the age of 83, and took it. She had brought up a large family, many of whom had passed through small-pox, and been attended by her, without her becoming affected with the disease. It has been said that an exemption from the disease prevails in particular families, but Dr. Gregory states that this idea is entirely without foundation.

That small-pox may occur a second time in the same individual is a well-known fact; but our author states it to be much less frequent than is supposed "At the Small-Pox Hospital very few present themselves who affirm that they have previously undergone small-pox; and of the few who do, but a very small portion can stand the test of a rigid scrutiny." Louis XV. of France is said to have died of a second attack of small-pox, but Dr. Gregory (who has examined the details carefully) is of opinion that the first attack was not really variola.

The power of medical treatment over small-pox is by no means so striking as in inflammation; nevertheless, remedies exert a certain degree of influence upon the disease, and the things to be attempted are thus enumerated

1. To moderate the violence of febrile excitement whenever we meet with it.

2. To check and relieve local determinations of blood, at whatever period of the disease they arise.

3. To support the powers of the system when they flag, either from the malignity of the poison or the long continuance of the disorder.

4. To combat, by appropriate means, concomitant disease.

One of the most interesting questions certainly connected with the treatment of small-pox relates to blood-letting. On this point Dr. Gregory makes the following observations:

"Some writers, in their zeal for blood-letting, have tried to persuade themselves that it is the only measure which can be relied on to lessen confluence, and to prevent the development of pustules in the mucous membrane of the throat and trachea. This opinion is altogether erroneous. Bleeding has no effect on the quantity of eruption, whether cutaneous or mucous. The most confluent eruption has succeeded to the most vigorous employment of the lancet. To bleed, therefore, merely because small-pox is anticipated, with the view, thereby, of preventing confluence, is uselessly to expend that power which will be required for the repair of injury to the surface. You will keep these general principles before you, aud take care that in your efforts to diminish internal congestion you do not materially impair constitutional power.

"If the stomach, during the initiatory fever, remains very irritable, rejecting everything that is taken, even the saline effervescing draughts with laudanum, which you will naturally feel disposed to try, I recommend you to apply mustard poultices to the epigastrium and to the feet, and to promote eruption by the pediluvium.

"Again: if the circulation at this period be languid, if the pulse be small and feeble, the skin pale, and the extremities cold; if the patient lies on his back, sunk and exhausted, let him have immediately warm brandy and water, cover him with bedclothes, apply mustard poultices to the centre and extremities of the circulating system, and give thirty drops of laudanum, to be repeated in four hours, if necessary. This cordial plan of treatment must often be continued for several days, when the eruptive nisus is accompanied with depression, and nature appears so obviously unequal to the effort."

In ordinary cases, the great object is to lessen the patient's suffering, without interrupting the process of pustulation; and, if there be nothing particular, a few saline draughts, and a little castor-oil, so as to keep the bowels free, are all that is required. The petechial form of the disease admits, he thinks, of no essential relief from medicine. "The loss of a little blood from the arm has appeared to me more effectual than any other measure." In all cases a warm bath is advisable before the patient again mixes in society.

Where, in the process of the secondary fever, apoplexy, peripneumony, or pleurisy comes on, blood may be taken freely; but in erysipelas, bark and wine are sometimes required. In ophthalmia (one of the most serious evils which follows small-pox) it is sometimes necessary to sacrifice the eye to save the patient's life—the state of whose system prohibits the requisite depletions.

In regard to the eruption itself, Dr. Gregory informs us that benefit is frequently derived from covering the surface with dry powder, such as starch; while fomentations and poultices are the only local means of any service in the abcesses and erythematous inflammations which sometimes attend the stage of secondary fever.

All the plans hitherto suggested for preventing pitting, have, in our author's hands, proved unavailing.

On the subject of inoculation, the opinions of our author are somewhat different from those entertained by most persons, and we shall conclude this part of our analysis by quoting his opinions in his own words:

"Since the introduction of vaccination, it has been the fashion to decry inoculation, and to impute to it mischief of which it was not guilty. The great objection made to inoculation, and that which recently induced Parliament to abolish it altogether, under heavy penalties, was, that it desseminated the virus, and multiplied the foci of contagion. Dr. Watkinsun and Dr. Schwenke, in 1777, and more recently, Dr. Adams, broke the force of this argument, by pointing out how important a part epidemic influence plays in the diffusion of variola. Had they lived in our times, how strongly would they have fortified their arguments! We saw, in 1838, an epidemic small-pox raging in London, where inoculation had long been discontinued. The admissions into the Small-Pox Hospital in that year exceeded those of 1781 and of 1796. Inoculation was abolished throughout England and Wales in 1840, and the act has been most rigidly enforced; yet, during the last two years, small-pox has visited every county of England.

"Sir Gilbert Blane has attempted to prove by statistics the evils of inoculation. He has shown that the proportion which the mortality by small-pox in London bore to the general mortality, increased during the last century from seventy-eight to ninety-four per thousand. But various considerations serve to weaken the force of this argument. If, for instance, we divide the last ninety years of the eighteenth century into three periods, we shall find that the recorded deaths by small-pox were as follow: 1711 to 1740 (when there was no inoculation), 55,383; 1741 to 1770 (when inoculation was coming into general use), 63,308; 1771 to 1800 (when inoculation was almost universal), the deaths were only 57,268; so that, by this showing, inoculation diminished the mortality by 8,115 lives!

"Statistics are very useful, and deservedly carry great weight with them; but they may be enlisted, with a little management, on both sides of an argument.

"One subject only remains for our consideration, and that is, the question, whether any circumstances would still warrant us in recommending inoculation on scientific principles? Concurring most cordially in opinion that the practice of inoculation by *unqualified* persons ought to have been put down (not in 1840, but forty years before that) by stringent legislative enactments, I still remain of opinion that under several circumstances it is the duty of a medical man to recommend inoculation. These circumstances do not, indeed, often occur; but the legislature would hardly wish to control and fetter, even in a single case, the deliberate judgment of a physician, acting for the benefit of his patient. I will name to you four of these cases: 1. When a person has been found, from peculiarity of habit, unsusceptible of vaccination. 2. When new sources of vaccine lymph are introduced, and it becomes of importance to ascertain that the new virus is efficient. 3. When young persons (between the ages of ten and twenty), vaccinated in early life, are proceeding as cadets to India. 4. When small-pox unexpectedly breaks out in a country district, at a time when (even with the facilities of a penny post) vaccine virus is not to be obtained.

"Other cases, equally strong, might be put; but what I have said will probably suffice to show that a clause (duly guarded against abuse) permitting qualified medical practitioners to inoculate under circumstances of urgency, would have been a useful addition to the Vaccination Extension Bill. That it was not so added was no fault of mine."—*Lon. Med. Gaz.*, Oct. 27, 1843.

